# Extraction of Pain Severity and Functional Interference From Clinical Narratives Using Domain-Informed Large Language Models: Protocol for a Development and Validation Study

**DOI:** 10.2196/96346

**Published:** 2026-10-07

**Authors:** Xiaoyi Zhang, Christopher R Wilson, Hannah Eyre, David E Reed II, Travis Y Hee Wai, Alexander Kloehn, Ethan W Rosser, Gang Luo, Steven B Zeliadt

**Affiliations:** 1 Department of Biomedical Informatics and Medical Education, University of Washington, Seattle, WA, United States; 2 Seattle-Denver Center of Innovation for Veteran-Centered and Value-Driven Care, VA Puget Sound Health Care System, 1660 S. Columbian Way, Seattle, WA, 98108, United States, 1 2062774175; 3 Department of Psychiatry and Behavioral Sciences, University of Washington, Seattle, WA, United States; 4 Department of Health Systems and Population Health, School of Public Health, University of Washington, Seattle, WA, United States

**Keywords:** chronic pain, natural language processing, electronic health records, large language models, patient-reported outcomes, knowledge distillation, Veterans, pain assessment

## Abstract

**Background:**

Chronic pain is a leading cause of disability and requires multidimensional assessment of pain intensity and functioning, yet electronic health records rarely capture these measures systematically. By contrast, surveys collecting patient-reported outcomes can assess pain over multiple dimensions but remain resource-intensive and difficult to scale for continuous population-level monitoring.

**Objective:**

The objective of this study is to develop and validate a domain-informed natural language processing framework to derive pain severity and functional interference outcomes from unstructured clinical narratives. We aim to demonstrate that natural language processing–derived outcomes can serve as a reliable, scalable surrogate for resource-intensive patient-reported surveys.

**Methods:**

This study uses a retrospective cohort of 3725 Veterans with chronic musculoskeletal pain initiating complementary and integrative health therapies across 18 Veterans Health Administration Whole Health Flagship sites (2021‐2023). The dataset encompasses longitudinal patient-reported outcome surveys serving as the benchmark, linked with unstructured clinical narratives from the Veterans Health Administration electronic health record. Guided by established psychometric instruments and subject matter expert (SME) input, we developed a seed lexicon and annotation guidelines to identify language distinguishing 3 pain domains: pain severity, interference with enjoyment of life, and interference with general activities. Preliminary large language model (LLM) prompting was used to identify 600 candidate encounters (200 per domain) from 6747 notes across 260 patients for SME annotation, forming a ground truth validation sample. Two candidate LLMs will be evaluated on this sample; the best-performing LLM will generate a large library of span-level annotations to train a scalable, lightweight language model. The study uses a 3-stage validation process: (1) documentation completeness of pain interference in clinical narratives against SME-annotated references; (2) inference accuracy of the LLM-as-annotator and the fine-tuned lightweight model against SME annotations across note-level classification and span-level localization; and (3) concordance between the lightweight model’s output and patient-reported pain, enjoyment, and general activity scores across a range of temporal windows.

**Results:**

As of July 2026, the cohort of 3725 Veterans has been identified and linked to clinical notes. The seed lexicon and annotation guidelines have been developed. Applying a developmental LLM to screen 6747 text notes in 6642 unique encounters over a 7-month period for 260 patients, at least 1 of the 3 pain domains was identified in 75% of notes and 99% of patients. SME validation at the encounter level is in progress. Final results from the subsequent knowledge distillation and validation stages are expected in the first half of 2027.

**Conclusions:**

This protocol outlines a framework for identifying severe pain intensity and interference from clinical narratives, addressing a critical gap in health care system surveillance. To our knowledge, this is the first study to validate clinical text-based pain outcome extraction against patient-reported outcomes in a nationwide longitudinal cohort. If successful, this approach will enable health care systems to continuously monitor reports of pain-related functional interference and support more holistic, patient-centered pain management at scale.

## Introduction

### Background

Chronic pain is a pervasive health issue that leads to poor patient outcomes and high health care costs. It affects one-fourth of adults in the United States, ranks as a leading cause of disability, and costs the nation over US $700 billion annually [1-3]. The prevalence and severity of chronic pain among Veterans of the US military are disproportionately high compared with the general population [4]. As such, chronic pain management is a high priority for health care providers and systems, including the Veterans Health Administration (VA) [5].

As pain is a subjective, multidimensional experience, its assessment is complex but central to evaluating treatment efficacy and effectiveness. The IMMPACT (Initiative on Methods, Measurement, and Pain Assessment in Clinical Trials) consensus recommends assessment of multiple core outcome domains for chronic pain treatment trials, including pain (with its intensity, quality, and temporal aspects), physical functioning, and emotional functioning [6,7]. Assessing these domains, whether capturing the subjective experience of pain severity or outcomes concerning pain’s interference with functioning (eg, general activity) and well-being (eg, enjoyment of life), relies heavily on patients’ self-reports.

While electronic health records (EHRs) are increasingly integrating self-reported pain data, with VA leading the shift from basic “fifth vital sign” scores [8] to comprehensive measures [9,10], systematic implementation of multidimensional patient-reported pain measures is rare [11]. The commonly used Numerical Rating Scale (NRS) for pain intensity, a single-item numeric measurement, was intended for clinical screening and has limited utility for longitudinally tracking outcomes or capturing pain’s broad interference in patient functioning and well-being [6,9,12-14]. Although multidimensional instruments like the Brief Pain Inventory, PEG (Pain, Enjoyment, and General Activity) scale, and Patient-Reported Outcomes Measurement Information System (PROMIS) measures offer richer insights, they are typically administered in specific research settings or specialty clinics rather than in routine care [13]. As pain is often discussed with providers as part of routine care, descriptions of pain interference, treatment plans, and associated progress may offer an alternative way to monitor the multidimensional aspects of pain management, supplementing targeted efforts to capture patient-reported pain outcomes.

### Prior Work

As part of a prior quality improvement initiative, VA conducted a nationwide survey across 18 VA medical centers participating in the Whole Health Flagship initiative, collecting patient-reported outcomes (PROs) from Veterans initiating complementary and integrative health (CIH) therapies [15,16]. The survey used measures from the PEG scale, a validated and widely used instrument to assess pain severity and its interference with functioning and quality of life [13]. Over the 2-year period spanning from March 2021 to March 2023, patient surveys were used to track longitudinal PROs from 3725 patients. Although providing valuable patient-reported data, surveys are resource-intensive, suffer from low response rates, and are difficult to scale for continuous population-level monitoring.

An alternative approach to obtaining outcomes equivalent to PROs at scale is through clinical notes, which contain narratives of patient reports and patient-provider discussions. Recent advances in machine learning and natural language processing (NLP) have generated techniques to extract pain and self-reported outcomes from narrative text data [17,18]. Prior works have leveraged NLP methods to achieve objectives such as constructing an ontology of pain over diverse attributes [19], identifying attributes of pain interference [20], and using fine-tuned large language models (LLMs) to parse musculoskeletal pain features from unstructured clinical notes [21]. Notably, recent studies using advanced Transformer-based deep learning models [22] found that model-extracted features of pain experience were in agreement with domain experts [20,23].

Despite the growing body of literature demonstrating the feasibility of using NLP to extract outcomes similar to PROs of chronic pain, several questions remain. First, documentation patterns for pain vary significantly across clinics and patient sociodemographic characteristics [24]. How such variation manifests within the heterogeneous VA population and whether it introduces systematic bias into algorithmic models remains largely unknown. Second, while the broad notion of pain is frequently documented, documentation of concepts related to subjective aspects such as patient-reported functioning is reportedly more sparse compared with aspects like severity or site [25,26]. A quantitative understanding of how completely clinical text data captures pain severity and functional interference domains remains underexamined. With these challenges in mind, we aim to develop a domain-informed NLP framework to operationalize these pain outcomes, leveraging linked longitudinal survey data as the benchmark to systematically quantify documentation completeness and validate the model’s clinical utility.

### Study Objectives

The primary objective of this study is to develop a domain-informed NLP framework for operationalizing pain severity and functional interference outcomes directly from clinical text data that can be scaled at the population level. Leveraging our survey dataset of 3725 Veterans with longitudinal PEG measures and an encounter with the VA within a year of the survey as the benchmark, we pursue 3 specific aims:

1. Quantify how completely EHR clinical notes capture the 3 PEG domains of pain intensity and functional interference among patients with chronic pain engaging in pain management therapies.
2. Evaluate the accuracy of a best-performing LLM and a scalable, lightweight language model in extracting pain intensity and interference domains against subject matter expert (SME) annotations.
3. Assess the concordance between model-derived pain assessments from EHR encounters and proximal patient-reported PEG scores.

To our knowledge, this is the first large-scale study to validate clinical text-based pain outcome extraction against patient-reported measures in a longitudinal nationwide survey. Ultimately, we aim to establish the feasibility of using this NLP framework as a scalable, automated alternative to resource-intensive surveys, enabling health care systems to longitudinally monitor severe pain intensity and interference at the population level.

## Methods

### Overview

This protocol outlines an ongoing study to develop and validate an NLP framework for identifying severe pain intensity and interference from clinical narratives across VA medical centers, using longitudinal PROs from a nationwide survey as the benchmark (Figure 1). At the time of writing, the project has finalized its methodological design and is currently in the model development phase. In the initial phase, we have completed the construct operationalization, translating PEG scale domains into a concrete seed lexicon derived from psychometrically validated instruments for use in developing annotation guidelines for identifying PEG-related concepts in clinical notes and rating each of the 3 PEG domains as either not severe (<7/10) or severe (≥7/10).

Currently, we are in the process of adapting LLMs within VA’s secure computing environment through prompt engineering, incorporating annotation guidelines to guide evidence extraction from clinical notes. Early LLM output has identified 600 candidate encounters (200 for each of the 3 pain domains) that are being verified by SMEs. This ground truth validation dataset will be used to assess the performance of 2 candidate LLMs. Although these resource-intensive LLMs are not currently scalable for reviewing EHR notes at the population level, this phase provides initial proof-of-concept validation. To enable scalable processing at the population level, we will adopt a knowledge distillation approach, using the best-performing LLM to generate a large library of span-level annotations that will be used to train a lightweight language model. This step will also allow transparency in reviewing which key language elements the LLM parses to determine severity levels within each of the 3 PEG domains. The lightweight model will then be assessed for performance relative to the best-performing LLM. Finally, we will compare the model-generated PEG domain ratings from EHR encounters to temporally adjacent PEG scores reported by patients to assess how closely EHR reports agree with PROs. Throughout these steps, we will conduct a 3-stage validation process to characterize the completeness of pain intensity and interference documentation in clinical narratives, evaluate the inference accuracy of the LLM-as-annotator and the lightweight model, and assess concordance between the lightweight model’s output and PROs captured independently from the EHR. The completed TRIPOD+AI (Transparent Reporting of a Multivariable Prediction Model for Individual Prognosis or Diagnosis and Artificial Intelligence) reporting checklist [27] is provided in Checklist 1.

**Figure 1.**
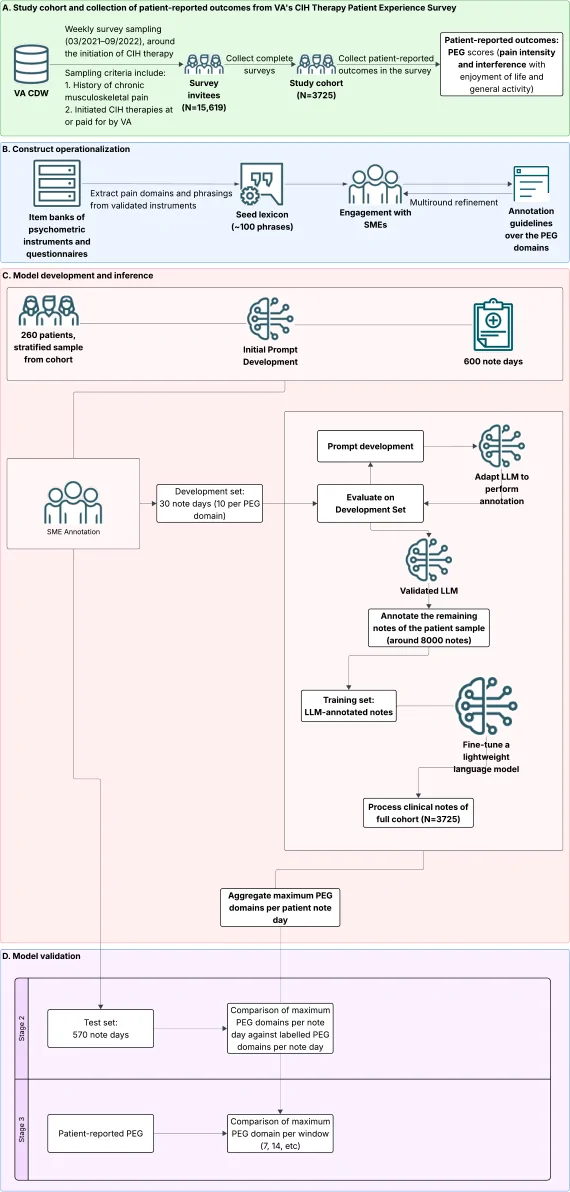
Overview of the NLP framework for identifying severe pain intensity and interference from clinical narratives. (A) Formation of the study cohort and collection of PEG scores as the benchmark. (B) Development of the seed lexicon and annotation guidelines. (C) Model development and inference, including LLM prompt refinement on SME-annotated notes, LLM annotation of the remaining patient sample, and fine-tuning of a lightweight language model applied to the full cohort. (D) Validation pipeline for both the LLM and lightweight language models. CDW: Corporate Data Warehouse; CIH: complementary and integrative health; LLM: large language model; NLP: natural language processing; PEG: pain, enjoyment, and general activity; SME: subject matter expert; VA: Veterans Health Administration.

### Ethical Considerations

This study constitutes a secondary analysis of PROs collected from the VA CIH Therapy Patient Experience Survey quality improvement evaluation [15,16]. This evaluation was deemed a nonresearch activity under the Federal Policy for the Protection of Human Subjects and was initiated and executed as an internal quality improvement project for VHA operations in accordance with VA Program Guide 1200.21 [28]. The present secondary analysis is conducted under an existing Memorandum of Understanding between the study team and the VA Office of Patient-Centered Care and Cultural Transformation, which governs data access for this work. No additional institutional review board review or waiver was required for this secondary analysis, as both the survey data from the initial evaluation project and the development of NLP evaluation tools to monitor pain interference in clinical narratives were deemed nonresearch.

### Data Governance and Privacy Safeguards

All analyses are conducted within VA’s Health Insurance Portability and Accountability Act (HIPAA)–compliant computing environment via the VA Informatics and Computing Infrastructure. All study data, including clinical notes, structured EHR fields, and survey responses, remain within VA firewalls at all times. Open-weight LLMs and the lightweight model are hosted on local VA graphics processing unit infrastructure, with no protected health information (PHI) transmitted to external API end points or third-party services; proprietary commercial LLMs accessed through external APIs are excluded from this study’s pipeline. Access to the underlying Corporate Data Warehouse is role-based and limited to personnel credentialed by the VA to conduct internal quality improvement data analytics, with all access events logged through VA Informatics and Computing Infrastructure’s standard auditing infrastructure.

### Study Setting

The Veterans Health Administration is the integrated health care system within the US Department of Veterans Affairs, providing care to enrolled Veterans through a national network of medical centers and outpatient sites. Enrollment is open to individuals with qualifying military service histories, although not all eligible Veterans enroll. Veterans receiving VA care carry a particularly heavy burden of chronic pain, with prevalence substantially higher than that of the general US adult population. This makes the cohort clinically important and represents a high-impact setting in which to develop scalable pain measurement tools. The Whole Health Flagship sites are 18 VA medical centers selected to lead implementation of VA’s Whole Health System of Care, a patient-centered health care model emphasizing what matters to the Veteran rather than what is the matter with the Veteran. The 6 priority CIH therapies expanded under this initiative are acupuncture, chiropractic care, massage, Tai Chi, yoga, and meditation.

Data for this study were derived from a secondary analysis of longitudinal PROs collected by Office of Patient-Centered Care and Cultural Transformation’s CIH Therapy Patient Experience Survey and corresponding EHRs from the VA Corporate Data Warehouse. The survey study protocol has been published [29,30]. VA’s Whole Health Flagship initiative, initially funded by the Comprehensive Addiction and Recovery Act of 2016, led to the expansion of CIH therapies across VA. The CIH Therapy Patient Experience Survey was conducted to assess the impact of this expansion on outcomes including pain severity and functional interference. Veterans invited to participate in the survey were aged 18 to 89 years, had been diagnosed with chronic musculoskeletal pain, and had newly initiated 1 of the 6 priority CIH therapies at any of the 18 VA medical centers participating in the Whole Health Flagship initiative.

Baseline surveys were sent on a weekly basis following identification of CIH therapy initiation between March 2021 and September 2022, with 6-month follow-up survey data collected through March 2023. Veterans included in this quality improvement evaluation were participants who had EHR-documented or self-reported use of a priority CIH therapy during the 6-month study period and who had completed self-reported pain assessment surveys at baseline and 6-month time points. This ensured that patients included in our study population had chronic pain and provided complete self-reported pain outcome data for analysis.

### Primary Pain Outcomes

The analysis focuses on replicating the individual domains of the PEG scale, a 3-item psychometric instrument consisting of 1 item for pain severity (“P”) and 2 items for pain interference with Enjoyment of life (“E”) and General activity (“G”) [13] (Figure 2). For each domain, the goal is to determine whether there is information in the clinical narrative deemed to represent a patient’s score of severe (≥7/10) or not severe (<7/10). The threshold of 7 for severe was determined from prior literature, which observed that this threshold explained the highest proportion of variance in patient-reported interference, as introduced by Serlin et al [31] and replicated in many different patient populations [32-34]. Future efforts may address methods for combining the 3 domains into a summary score and identifying finer-level gradients of pain interference; because the 3 PEG domains are certain to be documented at different frequencies in clinical narratives relative to survey collection, where all domains are structurally asked at the same time, combining the individual domains extracted from clinical narratives is a substantially different analytic task that will depend on the intermediate findings of this analysis.

**Figure 2.**
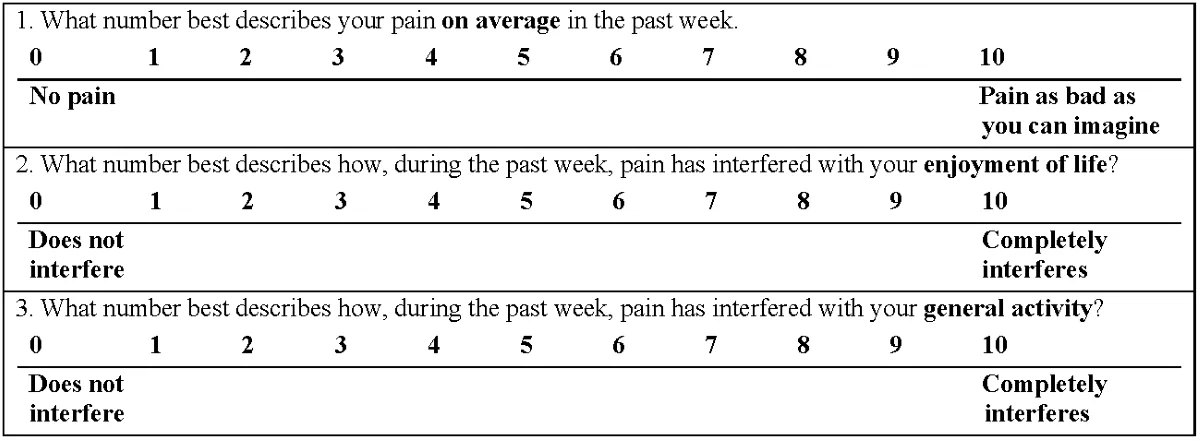
Pain, enjoyment, and general activity scale items administered in the complementary and integrative health Therapy Patient Experience Survey.

### Developing an NLP Pipeline to Identify Pain Severity and Interference From Clinical Notes

#### Overview and Rationale

Processing clinical notes at the population level requires a scalable approach. LLMs demonstrate strong performance in understanding clinical text, but their high memory and inference time requirements make direct application to hundreds of thousands of notes impractical within current local infrastructure constraints. To address this issue, we adopt a practical design inspired by knowledge distillation, with the goal of developing a scalable, lightweight language model that can process large volumes of notes. To ensure the lightweight language model can accurately accomplish the nuanced task of evidence extraction, we will develop a comprehensive library of reference annotations using a combination of human review and expansion using an LLM applied to a sample larger than what would be feasible for efficient human review.

#### Construct Operationalization

Pain interference is a multifaceted and abstract construct. Operationalizing this construct and extracting the related concrete information from clinical text present significant challenges. For instance, the PEG domain “Enjoyment of life” is a high-level concept that can be too abstract for direct extraction. To extract information relevant to pain intensity and interference as outlined in the PEG scale in a consistent and grounded manner, we developed an annotation guideline derived from multiple psychometric instruments, including item banks from the 41-item Patient-Reported Outcomes Measurement Information System-Pain Interference (PROMIS-PI) scale [35], the 52-item West Haven-Yale Multidimensional Pain Inventory (WHYMPI) [36], and the 10-item Oswestry Low Back Disability Questionnaire [37]. These instruments were selected to provide specific and comprehensive coverage across the PEG domains. For example, PEG’s “Enjoyment of life” (“E”) domain can be mapped to WHYMPI’s concepts of “satisfaction or enjoyment you get from participating in social and recreational activities.” Similarly, for PEG’s “General activity” (“G”) domain, the Oswestry Low Back Disability Questionnaire provides specific activity-based descriptions that align with documentation in clinical notes, such as “I can only walk using crutches or a cane” and “Pain prevents me from standing for more than 10 mins.” To capture PEG’s pain intensity domain (“P”), we also included explicit severity descriptors (eg, “severe pain,” “NRS 8/10”) commonly used in clinical narratives. This guideline serves as the foundation for SME annotation and as the supervision signal for our language models. We curated concepts and verbatims from these instruments to form a comprehensive lexicon of 101 pain descriptors, as listed in Multimedia Appendix 1.

#### Validation Sample Development

We are developing a validation sample of 600 encounters, targeting 200 encounters for each of the 3 P, E, and G domains, with at least 75 severe (≥7) encounters within each domain. To efficiently generate this validation corpus, we sampled 260 patients from the survey population and identified all unique notes occurring between 1 month prior to completion of the baseline survey and 6 months following completion. To ensure relevance, all notes were required to have at least 1 instance of the word “pain” to be sampled. After this restriction, these patients had 6747 notes across 6642 unique encounters during this period. An early LLM applied to the 6747 text notes preliminarily identified notes with severe and not severe descriptions of interference across the 3 pain domains (Table 1).

Using eHost software [38], annotation is being conducted by identifying sentence spans in clinical narratives that semantically map to concepts in the annotation guideline. Three SMEs with expertise in pain research are independently annotating clinical notes for a subset of patients sampled from the study population. Each SME reviews notes, identifies phrases representing PEG domains, and assigns severity to each domain using the annotation guide, grouped into “Not severe (0‐6)” and “Severe (7-10)” when appropriate descriptions are present in the text. Discrepancies are resolved through consensus adjudication among the SMEs.

**Table 1.** Preliminary counts of pain, enjoyment, and general activity domains and severity levels from the developmental large language model applied to the validation sample.

PEG^a^ domain and severity level	Unique patients (N=260), n (%)	Total notes over 7-month period (N=6747)^b^, n (%)
Any of 3 PEG domains identified	257 (99)	5092 (75)
P (pain severity)—any mention	257 (99)	4760 (71)
P (pain severity)—severe	165 (63)	848 (13)
E (interference with enjoyment of life)—any mention	187 (72)	960 (14)
E (interference with enjoyment of life)—severe	50 (19)	88 (1)
G (interference with general activities)—any mention	237 (91)	1938 (29)
G (interference with general activities)—severe	98 (38)	287 (4)

a PEG: pain, enjoyment, and general activity.

b Notes occurring on 6642 unique encounter dates.

#### Task Definition

Our pipeline involves 2 models, an LLM and a lightweight language model, each with distinct roles. After establishing proof of concept that the resource-intensive LLM can reliably identify elements of pain intensity and interference in clinical narratives, it will serve as an annotator (“LLM-as-Annotator”). Given the same information as provided to the SMEs, the LLM will receive free-text clinical narratives from our cohort. The expected output is extracted evidence mapped to the PEG domains, including relevant structured data when present within the note (eg, NRS scores) and specific sentence spans indicating pain intensity and interference, including markers of duration that represent severe interference such as “most days.” This will efficiently expand annotation capacity beyond what SMEs can achieve. An example input and annotation output is shown in Figure 3. These annotations will then serve as the supervision signal for fine-tuning the second model, a lightweight language model—such as a sentence transformer, a Bidirectional Encoder Representations from Transformers (BERT)–based architecture, or a lightweight LLM (<4B parameters)—that performs both evidence extraction and severe pain classification for the entire cohort. We plan to use ClinicalBERT for this task.

**Figure 3.**
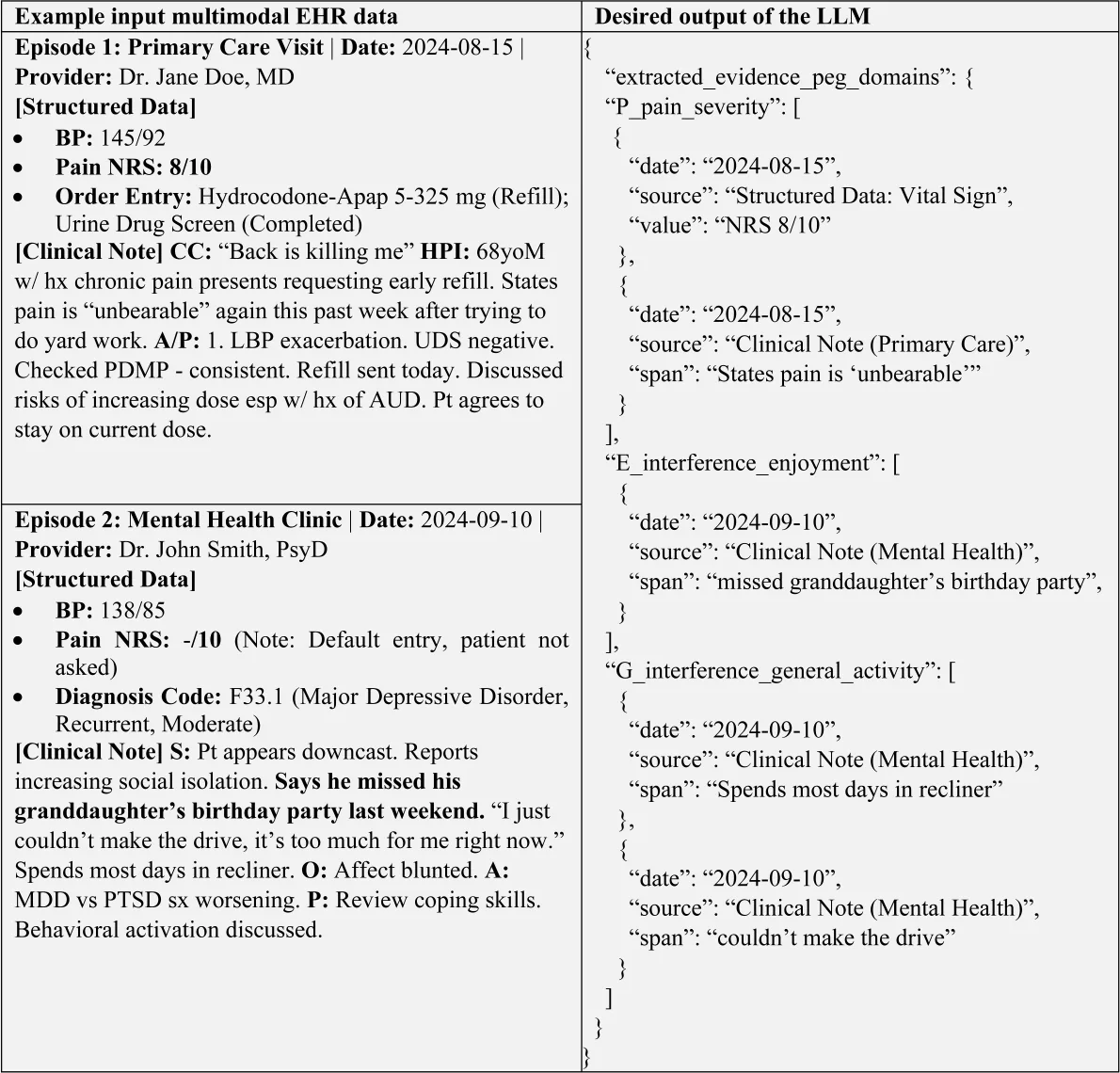
Output of the LLM-as-annotator for development of the annotation training library. AUD: alcohol use disorder; BP: blood pressure; CC: chief complaint; EHR: electronic health record; HPI: history of present illness; LBP: low back pain; LLM: large language model; MDD: major depressive disorder; NRS: numeric rating scale; PDMP: prescription drug monitoring program; PEG: pain, enjoyment of life, and general activity; Pt: patient; PTSD: posttraumatic stress disorder; sx: symptoms; UDS: urine drug screen.

#### Data Preprocessing

We will retrieve clinical notes anchored to the same temporal windows surrounding each survey time point. The data representation will be concatenated with the system prompt to the LLM, along with subsequent instructions for formatting the output.

#### Model Selection

We will leverage state-of-the-art open-weight (ie, models whose weights are publicly released, enabling local deployment) LLMs to perform this annotation task. Recent advances have demonstrated that LLMs pretrained on internet-scale corpora acquire extensive medical knowledge, with performance surpassing passing scores on professional medical examinations [39-42] and possessing robust medical reasoning capabilities [43]. For our application scenario, we will compare 2 open-weight models that are available within the secure VA computing environment and demonstrate strong performance on a variety of benchmarks: Gemma 4 E4B [44] and MedGemma 1.5 [45]. Comparison between the 2 candidate LLMs will be based on their agreement with SME annotations, as described in the Three-Stage Validation section below. These models will be hosted on local machines, ensuring that all computation remains within VA’s secure, HIPAA-compliant environment without transmitting PHI to external servers. For the downstream fine-tuned lightweight language model, we will evaluate lightweight architectures suitable for processing the entire cohort, as discussed in the *Knowledge Distillation for Scalable Inference* section below.

#### Adapting LLMs to Generate Annotations

The LLMs will be adapted through prompt engineering. Core definitions from our annotation guidelines will be used to develop the prompt, with a structured JSON output schema aligned with the PEG domains. As a baseline, we will evaluate zero-shot performance using only prompt instructions. To expose the model to real-world documentation patterns, we will implement few-shot prompting with up to 5 representative examples selected from our development annotation set. These examples will cover diverse clinical scenarios and documentation styles, each pairing raw clinical notes with reference JSON outputs containing extracted evidence.

To mitigate hallucination risk, we will deploy a chain-of-thought (CoT) output schema requiring a sequential reasoning method [46] where the model first identifies and quotes specific text spans indicating pain severity or functional interference, then maps this evidence to the corresponding PEG domains. By grounding outputs in cited evidence, the approach will enable systematic error analysis and iterative prompt refinement. This CoT reasoning is also important for complicated cases involving comorbidities. For example, social withdrawal documented in a mental health note may initially appear to stem from depression; however, if pain is documented elsewhere in the note as impacting the patient’s mood, this functional limitation should also be considered as pain attributable. By requiring the model to first extract all relevant evidence before making attributions, the CoT schema ensures such contextual information is accounted for. The transparency granted by CoT reasoning also facilitates SME validation of LLM outputs, as human experts can directly audit the extracted evidence and reasoning process.

#### Knowledge Distillation for Scalable Inference

To support scalable inference, we will adopt a knowledge distillation approach wherein LLM-generated annotations serve as the supervision signal for training a lightweight language model capable of processing the entire patient cohort. Our primary focus is ClinicalBERT [47], a domain-specific BERT-based architecture [48] pretrained on clinical and biomedical text, selected for its computational efficiency and its ability to be fully fine-tuned on local infrastructure to scale to population-level monitoring. Standard BERT-based models have a 512-token context limit, which may be insufficient for lengthy clinical notes. To address this, we will evaluate strategies including truncation to retain the most informative sections using rule-based sectionization found in medspaCy [49], as well as long-context encoder architectures such as Clinical ModernBERT [50], which supports up to 8192 tokens while maintaining the computational efficiency required for full-cohort processing.

Fine-tuning will be implemented via the Hugging Face Transformers library in an information extraction framework. Hyperparameters will be selected through k-fold cross-validation on the LLM-generated data, using *F*_1_-score as the focus metric, with early stopping to mitigate overfitting. The highest-performing hyperparameter configuration, as measured by *F*_1_-score, and the highest-performing overall configuration will be used to process all notes for the cohort, generating phrases containing PEG concepts and interference labels for all patients.

#### Computational Feasibility and Optimization

Our local graphics processing unit infrastructure (4 NVIDIA L40S, 48 GB) supports inference of models up to 70B parameters at 16-bit floating-point precision. For further memory optimization, we may additionally implement 4-bit quantization [51], which significantly reduces the memory footprint compared with full precision. This allows us to deploy all open-weight models on local machines, guaranteeing that all computation is contained entirely within the VA’s secure, HIPAA-compliant environment without transmitting PHI to external servers.

#### Three-Stage Validation for Our NLP Pipeline

##### Overview of 3-Stage Validation Process

To assess the reliability and clinical utility of our proposed approach, we will employ a 3-stage validation process. First, we will characterize the completeness of pain documentation in clinical narratives against SME-annotated references, quantifying how the 3 PEG domains are covered across notes, patients, and clinical settings. Second, we will evaluate the inference accuracy of the LLM-as-annotator, comparing 2 candidate LLMs (Gemma 4 E4B and MedGemma 1.5), and of the fine-tuned lightweight model against SME annotations across both note-level classification of pain domain and severity and span-level localization of pain-relevant text. Third, we will test the population-level utility of the fine-tuned lightweight model by assessing concordance between PROs captured contemporaneously and lightweight model classifications of proximal clinical narratives.

Stage 1 and Stage 2 validation activities will be conducted with the sample of 260 patients, along with their 600 sampled notes, who participated in the CIH Therapy Patient Experience Survey; Stage 3 will be conducted among the full sample of 3725 survey participants. The validation sample of 260 patients includes 6747 unique text notes that include the keyword “pain” over a 7-month period across 6642 unique encounters.

##### Stage 1: Documentation of Pain Intensity and Interference in Clinical Narratives

The first validation measures the extent to which EHR documentation can be assessed for meaningful elements of pain intensity and pain interference, using the targeted set of 600 SME-annotated notes identified from the preliminary LLM prompt as the basis for this analysis. As noted in Table 1, the frequency of PEG domains may vary considerably, with the E domain reported less frequently. The goal of this validation stage is to confirm that the core elements defined in the annotation guidelines and used by the LLMs are reliably identified in clinical narratives. The analysis unit for this stage is the full encounter day, with SMEs reviewing all notes from an encounter date to ensure all contextual information is assessed. The most severe intensity or interference level on the day will be assessed by the SME. This activity will provide additional training for both refinement of the LLM prompts and span-level annotation for fine-tuning of the lightweight model, as SMEs will be asked to highlight specific text spans they used in their judgment to assess each of the PEG domains. We define the following metrics:

- Interpretability: we report the proportion of notes in which pain was mentioned but SMEs could not determine domain or severity (“indeterminate”), and the proportion of spans requiring consensus adjudication.
- Interrater reliability: prior to any adjudication, agreement among the 3 SMEs will be quantified using Fleiss κ for the binary severity classification and for per-domain presence/absence. Because the sample is enriched for pain-relevant content by design, we note that these completeness metrics describe interpretable content within candidate notes rather than population-level documentation prevalence. Population-level documentation metrics will be assessed in subsequent validation stages using ClinicalBERT.

While preliminary examination of notes and our early LLM prompting have suggested there are extensive pain-related descriptions documented in clinical notes, this validation will shed light on potential gaps in EHR-documented clinical narratives. Potential limitations may arise due to limited provider documentation or insufficient descriptions by patients, leading to mentions of pain interference but uncertainty surrounding the extent of severity. To identify sources of bias prior to model deployment, we will examine how these metrics vary by clinical characteristics including age and sex.

In addition, we will record the proportion of notes in which a specific PEG domain is not mentioned as documentation gaps. Documentation gaps may arise from multiple sources, including limited symptom presence during the temporal window, provider documentation practices, and patient self-reporting patterns during clinical encounters. Our framework does not distinguish among these sources at the level of individual observations, as they collectively contribute to the observable evidence density from routine clinical documentation. Documentation gaps are characterized as observations of this evidence density rather than treated as missing data.

##### Stage 2: Inference Accuracy of the LLM-as-Annotator and Lightweight Model Against SME Annotation

###### LLM-as-Annotator Evaluation

Conditional upon documentation completeness established in stage 1, this stage evaluates whether an LLM can serve as a reliable annotator, extending SME annotation capacity to a scale suitable for training a downstream lightweight model. Two candidate LLMs, Gemma 4 E4B [44] and MedGemma 1.5 [45], will be evaluated against the 600 SME-annotated notes across 2 complementary sub-analyses: note-level classification and span-level localization.

###### Note-Level Classification of Domain and Severity

The purpose of this subanalysis is to demonstrate that an LLM can correctly identify descriptions of pain from text records and correctly classify its domain and severity. The LLM-as-annotator will be evaluated against the reference human annotations from the 600-encounter validation set to assess agreement with SME judgments (Multimedia Appendix 2). Each LLM’s performance will be calculated for each PEG domain and for binary level of severity if considered to contain elements suggestive of a level ≥7, to validate how it performs against each of the individual domains and overall intensity. Precision, recall, and *F*_1_-scores will be computed for each, with 95% CIs constructed by bootstrapping individuals and their corresponding notes, with replacement, to account for repeated measures within individuals (1000 replicates). An overall model-specific *F*_1_ measure will also be reported to compare the 2 candidate LLMs.

###### Span-Level Localization

Conditional on the note/visit-level analysis identifying an LLM that can accurately identify the key PEG domains and levels of severity from patient notes, this subanalysis evaluates whether the LLM-as-annotator can identify the specific span of text and assign the correct pain domain and severity label. The goal is to demonstrate that using the LLM to develop a large dataset of span annotations maintains a high quality of accuracy. This large training dataset supports development of the lightweight language model, which can then scale to the entire patient cohort (Figure 3).

We first evaluate whether the LLM extracts the same pain-relevant text spans as our human SMEs. Given the generative nature of LLMs, outputs may not be verbatim copies of source text. We will employ 2 matching criteria:

- Relaxed string match: A match is recorded if the extracted span shares tokens with the annotated span, relaxing exact boundary requirements.
- Semantic equivalence: We will use an LLM-as-a-judge approach, prompting a separate LLM to determine whether extracted content preserves the same meaning as the annotation. A recent study has shown that LLM-as-a-judge provides a scalable way to identify accurate and safe LLM-generated clinical summaries [52].

For both criteria, we will report precision, recall, and *F*_1_-score. Precision measures the proportion of LLM-extracted spans that overlap with reference annotations. Any nonoverlapping extractions that do not exist in the source text constitute hallucinations. Recall measures the proportion of reference annotations successfully retrieved by the model. CIs for each performance metric will be constructed by bootstrapping individuals in the validation set, with replacement, to account for repeated observations within individuals (1000 replicates). For each of the 3 PEG domains, we will compare spans identified by the LLM to the SME annotations and report the full confusion matrix along with per-class precision, recall, and *F*_1_ with bootstrapped 95% CIs. We will separately report the same metrics for the binary severity label (severe vs not severe) for each of the 3 domains.

Once the fine-tuned lightweight model is completed, we will repeat both note-level classification and span-level localization subanalyses using the lightweight model against the same 600 SME-annotated encounters. This provides direct evaluation of the lightweight model’s inference accuracy against the SME reference, alongside the LLM comparison.

### Stage 3: Concordance Between the Scalable Lightweight Model and PROs

The lightweight model is designed for scalable, population-level deployment rather than individual-level diagnostic classification. Accordingly, the evaluation of concordance is framed around whether the model’s output can serve as a reliable surrogate for PROs at population scale, complementing rather than replacing direct patient self-report. As such, we will assess the concordance between severe pain and interference identified by the lightweight model in clinical narratives and patient-reported PEG scores in the full 3725-patient cohort, with patient-reported scores serving as the benchmark (Multimedia Appendix 3). This analysis will be performed on the baseline data for each individual.

The primary discrimination metric is area under the receiver operating characteristic curve, computed individually for each PEG domain using the proportion of severe pain and interference notes within a survey-anchored window as the continuous ranking score against the patient’s binary per-domain survey label (severe ≥7/10 vs not severe <7/10).

Patients without any pain-related clinical narrative within a given temporal window are excluded from that window’s analysis, as no narrative observation exists. This exclusion is reflected in the reduced sample size at narrower windows (Multimedia Appendix 3). Patients with pain-related narratives but without documentation of a specific PEG domain are retained as observations of low evidence density for that domain.

In addition to discrimination, we will assess calibration to evaluate how closely model-predicted rates of severe pain interference align with patient-reported rates at the aggregate level. Calibration will be reported through 3 complementary measures. Calibration plots will bin patients by decile of predicted probability and compare predicted rates against observed patient-reported rates within each bin. Brier scores will summarize overall probabilistic performance across the full cohort, with lower values indicating closer agreement between predicted and observed outcomes. Calibration-in-the-large will be computed as the ratio of predicted to observed severe pain interference prevalence, providing a single-number summary of systematic overprediction or underprediction.

Each analysis will be repeated for a range of temporal windows around the survey date (±7, ±30, ±90, and ±180 d) to assess how the breadth of the aggregation window affects concordance. The 7-day window reflects the anchoring period of the PEG instrument, which asks patients to consider the past week. All point estimates will be reported with 95% CIs derived from a nonparametric bootstrap resampling patients as the independent unit over 1000 replicates.

Given the model’s intended role as a population-scale complement to represent a similar concept as patient self-report of pain severity and interference, the following minimum performance criterion is calibrated to surrogate utility rather than individual-level diagnostic accuracy. The prespecified minimum performance criterion goal for Stage 3 is area under the receiver operating characteristic curve ≥0.75 for each of the 3 domains, requiring discrimination meaningfully above chance while accommodating the attenuation expected when validating an EHR-derived surrogate against patients’ self-reports. We anticipate performance will deteriorate as encounters/clinical narratives become less temporally related to the survey date.

Performance will additionally be evaluated within prespecified subgroups of interest (age, sex, primary pain diagnosis, and clinic type) to determine whether the model systematically overestimates or underestimates severe pain interference for these subgroups. Subgroup differences will be assessed by comparing bootstrap-derived confidence intervals across subgroups.

Together, the 3 validation stages assess the full pipeline from documentation completeness to model inference accuracy and population-level utility. These results will determine the feasibility of monitoring severe pain and interference at population scale and whether our NLP framework can serve as a scalable surrogate for resource-intensive patient-reported surveys.

## Results

This study is ongoing, with final results expected in 2027. As of July 2026, the study cohort of 3725 Veterans with chronic musculoskeletal pain who were new users of CIH therapies by survey administration date has been identified. The longitudinal PEG survey data serving as the benchmark have been fully aggregated. Demographic and clinical characteristics of the cohort have been previously published [53].

The domain-specific seed lexicon has been developed, curating 101 pain descriptors from established psychometric instruments including PROMIS-PI, WHYMPI, and the Oswestry Low Back Disability Questionnaire. Annotation guidelines mapping these concepts to each of the 3 PEG domains have been developed and are being iteratively refined through engagement with SMEs. We have applied a developmental LLM to screen 6747 text notes among 6642 unique encounters over a 7-month period for 260 patients, with the preliminary LLM identifying at least 1 of the 3 PEG domains in 75% of notes and 99% of patients (Table 1). Validation at the note level, targeting 200 notes for each of the 3 PEG domains, is in progress as of July 2026. Upon completion of the validation-sample annotation, the study will proceed to the subsequent knowledge distillation and validation stages.

## Discussion

### Principal Implications

Chronic pain is a complex health issue. Current screening tools embedded in structured EHR data cannot fully capture this complexity. Our framework extracts multidimensional pain information, including pain severity and interference with physical and emotional functioning, directly from clinical narratives at scale. If validated, this approach could offer a scalable alternative to resource-intensive surveys for monitoring severe pain and interference. To the best of our knowledge, this is the first study to investigate the feasibility of using EHR data, validated against large-scale PROs, for routine monitoring of severe pain and interference.

### Methodological Significance

This study adopts a domain-informed approach, grounding the LLM’s inference in a seed lexicon derived from established psychometric instruments. This enables rigorous mapping of abstract pain-related PEG domains into concrete terms found in clinical documentation. To enable scalable processing, our approach employs knowledge distillation: an LLM first generates high-quality annotations on a subset of notes, which then serve as training data for a lightweight language model capable of processing the entire cohort. This design leverages LLM capabilities for nuanced evidence extraction while enabling scalable inference through a lightweight model. Our validation strategy extends beyond standard benchmarking against expert annotations. By first examining documentation completeness, we will assess whether disparities in clinical documentation practices, which have been identified in prior work [24-26] and may introduce systemic bias, exist in our patient population. By validating against PROs, we will evaluate whether the framework’s inferences align with how patients themselves report their pain and functional interference.

### Clinical and Operational Utility

Subject to successful validation, this framework could improve how pain is monitored in health care systems. Surveys collecting PROs often suffer from low response rates and nonresponse bias, as prior research found that nonresponders had higher pain-catastrophizing scores and more pain at enrollment [12]. A validated NLP tool could serve as a passive approach to identify severe pain and functional interference, complementing active survey collection and reaching patients who are engaged in care but unreached by surveys. A validated severe pain phenotype would also enable longitudinal monitoring of treatment outcomes. While randomized controlled trials establish efficacy, they are limited in tracking long-term functional improvements at scale. By extracting pain and functional interference directly from EHR data, researchers could evaluate the efficacy of high-cost or invasive interventions such as spinal cord stimulation in reducing severe pain and interference in broader, real-world populations.

### Limitations

There are several limitations to this protocol. First, the LLMs’ inference relies on EHR documentation, which may be incomplete in recording the full picture of patients’ experience of pain. We address this limitation by quantifying documentation completeness in our validation. Second, pain fluctuates over time, and temporal gaps between clinical visits and survey dates may reduce concordance between EHR-derived and survey-reported outcomes. Third, significant heterogeneity in clinical documentation practices and reports of interference from pain will likely lead to underidentification of interference. Fourth, documentation biases in the EHR may propagate into model inference. If pain is systematically underdocumented for certain patient populations, the language model’s inference for those groups may inherit this bias. We will conduct subgroup analyses by demographic and socioeconomic characteristics, comparing both documentation patterns (eg, the frequency and coverage of the PEG domains in annotations) and model inference accuracy metrics (eg, sensitivity, concordance with PROs) across groups to identify such disparities.

Fifth, patient representatives were not directly consulted during the development of the seed lexicon, annotation guidelines, or interpretation of model outputs in this secondary analysis, as the parent CIH Therapy Patient Experience Survey was conducted between 2021 and 2023 and has since concluded. This gap is partially mitigated by the fact that patient perspectives are embedded indirectly through the psychometric instruments used to construct the seed lexicon (PROMIS-PI, WHYMPI, Oswestry), each of which was developed with patient-engaged validation. Nonetheless, future deployment work would benefit from direct patient input on interpretability and face validity.

Finally, the direct comparison between aggregated NLP-derived domain evidence and PEG survey scores is an imperfect proxy for true concordance. Clinical documentation of pain is episodic and contextually driven, whereas the PEG reflects a patient’s deliberate summary of their experience over time; simple aggregation of sparse span-level extractions may not fully bridge this representational gap. A natural extension of this work, contingent on demonstrating adequate NLP accuracy against SME annotations, would be to train a supervised model that learns to map heterogeneous, potentially sparse clinical documentation patterns onto PEG-equivalent scores, rather than relying on direct aggregation.

### Conclusion

This protocol outlines a framework for identifying severe pain intensity and interference from clinical narratives, addressing a gap in health care system surveillance. By grounding inference in established psychometric instruments and validating against PROs, we aim to develop an NLP-empowered tool that can reliably extract pain severity and functional interference from EHR data. If successful, this approach could provide a scalable complement to resource-intensive surveys, enabling longitudinal monitoring of severe pain interference at the population level.

## Supplementary material

10.2196/96346 Multimedia Appendix 1 Seed lexicon of 101 terms derived from validated pain assessment instruments and mapped to the 3 PEG domains (Pain severity, Enjoyment of life, General activity).

10.2196/96346 Multimedia Appendix 2 Shell table—encounter-level classification accuracy of candidate large language models against subject matter expert annotation for each pain severity, enjoyment of life, general activity domain and for binary severity level ≥7.

10.2196/96346 Multimedia Appendix 3 Shell table (partial)—final validation reporting of Lightweight Model and survey responses.

10.2196/96346 Checklist 1 TRIPOD+AI checklist.

## Abbreviations

BERT: Bidirectional Encoder Representations from Transformers
CDW: Corporate Data Warehouse
CIH: complementary and integrative health
CoT: chain-of-thought
EHR: electronic health record
HIPAA: Health Insurance Portability and Accountability Act
IMMPACT: Initiative on Methods, Measurement, and Pain Assessment in Clinical Trials
LLM: large language model
NLP: natural language processing
NRS: Numerical Rating Scale
PEG: pain, enjoyment, and general activity
PHI: protected health information
PRO: patient-reported outcome
PROMIS-PI: Patient-Reported Outcomes Measurement Information System-Pain Interference
SME: subject matter expert
TRIPOD+AI: Transparent Reporting of a Multivariable Prediction Model for Individual Prognosis or Diagnosis and Artificial Intelligence
VA: Veterans Health Administration
WHYMPI: West Haven-Yale Multidimensional Pain Inventory
