# Supplementary material for: Extraction of Pain Severity and Functional Interference From Clinical Narratives Using Domain-Informed Large Language Models: Protocol for a Development and Validation Study
Source: JMIR Res Protoc. 2026 Oct 7;15:e96346. doi: 10.2196/96346 (PMC13645315; doi:10.2196/96346)
Supplement: Supplementary file 1 [file resprot-v15-e96346-s001.pdf]

No.	PEG Domain	Seed Lexicon Terms	Source Instrument(s)	Source Item(s)
1	P: Pain Severity	pain medication provides certain relief	Oswestry	S1: Pain medication provides me complete/moderate/little relief from pain
2	P: Pain Severity	pain medication has no effect	Oswestry	S1: Pain medication has no effect on the pain
3	P: Pain Severity	pain so severe could think of nothing else	PROMIS-PI	PI29: How often was your pain so severe you could think of nothing else?
4	P: Pain Severity	pain feels like a burden	PROMIS-PI	PI20: How much did pain feel like a burden to you?
5	P: Pain Severity	distressed by pain	PROMIS-PI	PI24: How often was pain distressing to you?
6	P: Pain Severity	pain level at present moment	WHYMPI	A1: Rate the level of your pain at the present moment
7	P: Pain Severity	very intense pain	WHYMPI	A1 answer: Very intense pain
8	P: Pain Severity	severe pain during the past week	WHYMPI	A7: How severe has your pain been during the last week?
9	P: Pain Severity	extremely severe pain	WHYMPI	A7 answer: Extremely severe
10	P: Pain Severity	suffering because of pain	WHYMPI	A12: How much suffering do you experience because of your pain?
11	P: Pain Severity	extreme suffering from pain	WHYMPI	A12 answer: Extreme suffering
12	E: Enjoyment of Life	social life increases pain	Oswestry	S8: My social life is normal, but it increases my level of pain
13	E: Enjoyment of Life	pain prevents participating in energetic activities	Oswestry	S8: Pain prevents me from participating in more energetic activities (sports, dancing)
14	E: Enjoyment of Life	pain prevents going out often	Oswestry	S8: Pain prevents me from going out very often
15	E: Enjoyment of Life	hardly any social life because of pain	Oswestry	S8: I have hardly any social life because of my pain
16	E: Enjoyment of Life	depressed due to pain	PROMIS-PI	PI16: How often did pain make you feel depressed?
17	E: Enjoyment of Life	anxious due to pain	PROMIS-PI	PI37: How often did pain make you feel anxious?
18	E: Enjoyment of Life	irritable because of pain	PROMIS-PI	PI56: How irritable did you feel because of pain?
19	E: Enjoyment of Life	discouraged by pain	PROMIS-PI	PI32: How often did pain make you feel discouraged?
20	E: Enjoyment of Life	emotionally tense because of pain	PROMIS-PI	PI11: How often did you feel emotionally tense because of your pain?
21	E: Enjoyment of Life	pain interferes with enjoyment of life	PROMIS-PI	PI3: How much did pain interfere with your enjoyment of life?
22	E: Enjoyment of Life	pain interferes with things usually done for fun	PROMIS-PI	PI12: How much did pain interfere with the things you usually do for fun?
23	E: Enjoyment of Life	pain interferes with enjoyment of recreational activities	PROMIS-PI	PI10: How much did pain interfere with your enjoyment of recreational activities?
24	E: Enjoyment of Life	pain interferes with enjoyment of social activities	PROMIS-PI	PI36: How much did pain interfere with your enjoyment of social activities?
25	E: Enjoyment of Life	avoids social activities due to pain	PROMIS-PI	PI38: How often did you avoid social activities because it might make you hurt more?
26	E: Enjoyment of Life	pain keeps from socializing	PROMIS-PI	PI26: How often did pain keep you from socializing with others?

27	E: Enjoyment of Life	difficult to plan social activities	PROMIS-PI	PI46: How often did pain make it difficult for you to plan social activities?
28	E: Enjoyment of Life	can't plan activities due to unpredictable pain	PROMIS-PI	PI52: Hard to plan social activities because didn't know if you would be in pain
29	E: Enjoyment of Life	pain interferes with participation in social activities	PROMIS-PI	PI31: How much did pain interfere with ability to participate in social activities?
30	E: Enjoyment of Life	pain interferes with leisure activities	PROMIS-PI	PI5: How much did pain interfere with ability to participate in leisure activities?
31	E: Enjoyment of Life	pain interferes with close personal relationships	PROMIS-PI	PI6: How much did pain interfere with your close personal relationships?
32	E: Enjoyment of Life	pain interferes with relationships with other people	PROMIS-PI	PI17: How much did pain interfere with your relationships with other people?
33	E: Enjoyment of Life	pain interferes with family life	PROMIS-PI	PI13: How much did pain interfere with your family life?
34	E: Enjoyment of Life	social life restricted to home	PROMIS-PI, Oswestry	PI53: How often did pain restrict your social life to your home? / S8: Pain has restricted my social life to my home
35	E: Enjoyment of Life	overall mood extremely low	WHYMPI	A6: Rate your overall mood during the past week
36	E: Enjoyment of Life	irritable	WHYMPI	A18: During the past week, how irritable have you been?
37	E: Enjoyment of Life	tense or anxious	WHYMPI	A20: During the past week, how tense or anxious have you been?
38	E: Enjoyment of Life	not able to deal with problems	WHYMPI	A16: How much do you feel you've been able to deal with your problems?
39	E: Enjoyment of Life	not in control of life	WHYMPI	A11: How much control do you feel you have had over your life?
40	E: Enjoyment of Life	reduced satisfaction from social and recreational activities	WHYMPI	A4: How much has pain changed satisfaction from social and recreational activities?
41	E: Enjoyment of Life	reduced ability to participate in recreational and social activities	WHYMPI	A8: How much has pain changed your ability to participate in recreational and other social activities?
42	E: Enjoyment of Life	reduced satisfaction from family-related activities	WHYMPI	A9: How much has pain changed satisfaction from family-related activities?
43	E: Enjoyment of Life	reduced satisfaction from work	WHYMPI	A14: How much has pain changed satisfaction or enjoyment from work?
44	E: Enjoyment of Life	no longer goes out to eat	WHYMPI	C3: Go out to eat
45	E: Enjoyment of Life	no longer plays cards or games	WHYMPI	C4: Play cards or other games
46	E: Enjoyment of Life	no longer goes to movies	WHYMPI	C7: Go to a movie
47	E: Enjoyment of Life	no longer visits friends	WHYMPI	C8: Visit friends
48	E: Enjoyment of Life	no longer visits relatives	WHYMPI	C12: Visit relatives
49	E: Enjoyment of Life	no longer goes to park or beach	WHYMPI	C16: Go to a park or beach
50	E: Enjoyment of Life	pain has changed marriage and family relationships	WHYMPI	A13: How much has pain changed your marriage and other family relationships?

51	E: Enjoyment of Life	pain has changed friendships	WHYMPI	A19: How much has pain changed your friendships with people other than your family?
52	G: General Activity	personal care increases pain	Oswestry	S2: I can take care of myself normally but it increases my pain
53	G: General Activity	personal care is painful and slow	Oswestry	S2: It is painful to take care of myself and I am slow and careful
54	G: General Activity	needs help with personal care	Oswestry	S2: I need help but I am able to manage most of my personal care / I need help every day in most aspects of my care
55	G: General Activity	does not get dressed, stays in bed, can't do personal care due to pain	Oswestry	S2: I do not get dressed, wash with difficulty and stay in bed
56	G: General Activity	can only walk with crutches or a cane	Oswestry	S4: I can only walk using crutches or a cane
57	G: General Activity	in bed most of the time	Oswestry	S4: I am in bed most of the time and have to crawl to the toilet
58	G: General Activity	standing increases pain	Oswestry	S6: I can stand as long as I want but increases my pain
59	G: General Activity	pain prevents standing at all	Oswestry	S6: Pain prevents me from standing at all
60	G: General Activity	can only sit in favorite chair	Oswestry	S5: I can only sit in my favorite chair as long as I like
61	G: General Activity	pain prevents sitting at all	Oswestry	S5: Pain prevents me from sitting at all
62	G: General Activity	can sleep well only by using pain medication	Oswestry	S7: I can sleep well only by using pain medication
63	G: General Activity	reduced sleep duration even with medication	Oswestry	S7: Even when I take pain medication, I sleep less than 6/4/2 hours
64	G: General Activity	pain prevents sleeping at all	Oswestry	S7: Pain prevents me from sleeping at all
65	G: General Activity	pain makes it difficult to fall asleep	PROMIS-PI	PI19: How much did pain make it difficult to fall asleep?
66	G: General Activity	lifting increases pain	Oswestry	S3: I can lift heavy weights but it causes increased pain
67	G: General Activity	pain limits lifting	Oswestry	S3: Pain prevents me from lifting heavy weights off the floor / I can manage light to medium weights / I can lift only very light weights
68	G: General Activity	cannot lift or carry anything due to pain	Oswestry	S3: I cannot lift or carry anything at all
69	G: General Activity	normal job activities increase pain	Oswestry	S10: My normal homemaking/job activities increase my pain, but I can still perform all that is required
70	G: General Activity	pain limits to light duties only	Oswestry	S10: I can perform most duties but not physically stressful activities / Pain prevents me from doing anything but light duties
71	G: General Activity	pain prevents even light duties	Oswestry	S10: Pain prevents me from doing even light duties
72	G: General Activity	pain prevents any job or homemaking chores	Oswestry	S10: Pain prevents me from performing any job/homemaking chores
73	G: General Activity	pain restricts travel duration	Oswestry	S9: Pain restricts travel over 2 hours / over 1 hour / to short necessary journeys under 1/2 hour
74	G: General Activity	pain prevents all travel except hospital visits	Oswestry	S9: Pain prevents all travel except for visits to the doctor/therapist or hospital

75	G: General Activity	pain limits walking distance	Oswestry, PROMIS-PI	S4: Pain prevents me walking more than 1 mile / 1/2 mile / 1/4 mile; PI40: Pain prevent you from walking more than 1 mile
76	G: General Activity	pain limits ability to stand	Oswestry, PROMIS-PI	S6: Pain prevents me from standing for more than 1 hour / 30 min / 10 min; PI42/PI47
77	G: General Activity	pain limits ability to sit	Oswestry, PROMIS-PI	S5: Pain prevents me sitting more than 1 hour / 30 min / 10 min; PI55/PI50/PI51
78	G: General Activity	pain makes simple tasks hard to complete	PROMIS-PI	PI39: How often did pain make simple tasks hard to complete?
79	G: General Activity	pain interferes with work around the home	PROMIS-PI	PI22: How much did pain interfere with work around the home?
80	G: General Activity	pain interferes with tasks away from home	PROMIS-PI	PI14: How much did pain interfere with doing tasks away from home (groceries, errands)?
81	G: General Activity	pain keeps from getting into standing position	PROMIS-PI	PI54: How often did pain keep you from getting into a standing position?
82	G: General Activity	pain interferes with ability to work	PROMIS-PI	PI18: How much did pain interfere with your ability to work (include work at home)?
83	G: General Activity	pain interferes with trips from home over 2 hours	PROMIS-PI	PI35: How much did pain interfere with trips from home that kept you gone for more than 2 hours?
84	G: General Activity	pain interferes with ability to concentrate	PROMIS-PI	PI8: How much did pain interfere with your ability to concentrate?
85	G: General Activity	pain interferes with ability to remember things	PROMIS-PI	PI49: How much did pain interfere with your ability to remember things?
86	G: General Activity	difficult to take in new information because of pain	PROMIS-PI	PI1: How difficult was it for you to take in new information because of pain?
87	G: General Activity	pain interferes with day to day activities	PROMIS-PI, WHYMPI	PI9 / A2: How much did pain interfere with your day to day activities?
88	G: General Activity	pain interferes with household chores	PROMIS-PI, WHYMPI	PI34/PI48 / A17: How much did pain interfere with household chores?
89	G: General Activity	no longer washes dishes	WHYMPI	C1: Wash dishes
90	G: General Activity	no longer mows the lawn	WHYMPI	C2: Mow the lawn
91	G: General Activity	no longer goes grocery shopping	WHYMPI	C5: Go grocery shopping
92	G: General Activity	no longer works in the garden	WHYMPI	C6: Work in the garden
93	G: General Activity	no longer helps with house cleaning	WHYMPI	C9: Help with the house cleaning
94	G: General Activity	no longer works on the car	WHYMPI	C10: Work on the car
95	G: General Activity	no longer prepares meals	WHYMPI	C13: Prepare a meal
96	G: General Activity	no longer washes the car	WHYMPI	C14: Wash the car
97	G: General Activity	no longer does laundry	WHYMPI	C17: Do a load of laundry
98	G: General Activity	no longer does house repairs	WHYMPI	C18: Work on a needed house repair
99	G: General Activity	pain changed ability to work	WHYMPI	A3: How much has your pain changed your ability to work?

100	G: General Activity	no longer takes trips	WHYMPI	C15: Take a trip
101	G: General Activity	no longer takes rides in a car	WHYMPI	C11: Take a ride in a car
