# Supplementary material for: Extraction of Pain Severity and Functional Interference From Clinical Narratives Using Domain-Informed Large Language Models: Protocol for a Development and Validation Study
Source: JMIR Res Protoc. 2026 Oct 7;15:e96346. doi: 10.2196/96346 (PMC13645315; doi:10.2196/96346)
Supplement: Supplementary file 2 [file resprot-v15-e96346-s004.pdf]

Section/Topic	Item	Dev/Eval	Checklist item	Reported on sections
**TITLE**				
Title	1	D;E	Identify the study as developing or evaluating the performance of a multivariable prediction model, the target population, and the outcome to be predicted	Title page
**ABSTRACT**				
Abstract	2	D;E	See TRIPOD+AI for Abstracts checklist	Abstract section
**INTRODUCTION**				
Background	3a	D;E	Explain the healthcare context (including whether diagnostic or prognostic) and rationale for developing or evaluating the prediction model, including references to existing models	Introduction: Background; Prior Work
	3b	D;E	Describe the target population and the intended purpose of the prediction model in the context of the care pathway, including its intended users	Introduction: Background; Discussion: Clinical and Operational Utility
	3c	D;E	Describe any known health inequalities between sociodemographic groups	Introduction: Prior Work; Methods: Three-stage Validation (subgroup analyses); Discussion: Limitations
Objectives	4	D;E	Specify the study objectives, including whether the study describes the development or validation of a prediction model (or both)	Introduction: Study Objectives
**METHODS**				
Data	5a	D;E	Describe the sources of data separately for the development and evaluation datasets, the rationale for using these data, and representativeness of the data	Methods: Study Setting
	5b	D;E	Specify the dates of the collected participant data, including start and end of participant accrual; and, if applicable, end of follow-up	Methods: Study Setting
Participants	6a	D;E	Specify key elements of the study setting (e.g., primary care, secondary care, general population) including the number and location of centers	Methods: Study Setting
	6b	D;E	Describe the eligibility criteria for study participants	Methods: Study Setting
	6c	D;E	Give details of any treatments received, and how they were handled during model development or evaluation, if relevant	N/A. Study is observational; no treatments are administered as part of the study. CIH therapy initiation defines cohort eligibility and is described in Methods: Study Setting.
Data preparation	7	D;E	Describe any data pre-processing and quality checking, including whether this was similar across relevant sociodemographic groups	Methods: Data Pre-processing; Construct Operationalization via Human Annotation of Clinical Texts
Outcome	8a	D;E	Clearly define the outcome that is being predicted and the time horizon, including how and when assessed, the rationale for choosing this outcome, and whether the method of outcome assessment is consistent across sociodemographic groups	Methods: Primary Pain Outcomes
	8b	D;E	If outcome assessment requires subjective interpretation, describe the qualifications and demographic characteristics of the outcome assessors	N/A. Outcome (PEG domain score ≥ 7) is derived from patient self-report on a validated psychometric instrument;

Section/Topic	Item	Dev/Eval	Checklist item	Reported on sections
				no subjective assessor interpretation is involved.
	8c	D;E	Report any actions to blind assessment of the outcome to be predicted	Methods: Construct Operationalization via Human Annotation of Clinical Texts; Three-stage Validation (SME annotators blinded to PEG survey responses during note annotation)
Predictors	9a	D	Describe the choice of initial predictors and any pre-selection of predictors before model building	Methods: Data Pre-processing; Task Definition
	9b	D;E	Clearly define all predictors, including how and when they were measured (and any actions to blind assessment of predictors for the outcome and other predictors)	Methods: Construct Operationalization via Human Annotation of Clinical Texts; Data Pre-processing; Task Definition
	9c	D;E	If predictor measurement requires subjective interpretation, describe the qualifications and demographic characteristics of the predictor assessors	Methods: Construct Operationalization via Human Annotation of Clinical Texts (SME pain research expertise, annotation guideline development, iterative refinement, inter-rater reliability)
Sample size	10	D;E	Explain how the study size was arrived at (separately for development and evaluation), and justify that the study size was sufficient	Methods: Study Setting (n = 3,726 cohort); Construct Operationalization (260 patients / 600 notes for SME annotation); Training, model selection, and held-out evaluation (80/20 split). All available data are used; no formal a priori sample size calculation, as this is a fixed parent cohort.
Missing data	11	D;E	Describe how missing data were handled. Provide reasons for omitting any data	The survey sample by definition has complete PEG information for baseline responders and all participants have encounters within VA with possible clinical narratives. NA at protocol stage; missing data handling strategy to be finalized during model implementation. Discussion: Limitations
Analytical methods	12a	D	Describe how the data were used (e.g., for development and evaluation of model performance) in the analysis, including whether the data were partitioned, considering any sample size requirements	NA at protocol stage; data partitioning strategy to be finalized during model implementation

Section/Topic	Item	Dev/Eval	Checklist item	Reported on sections
	12b	D	Depending on the type of model, describe how predictors were handled in the analyses (functional form, rescaling, transformation, or any standardisation)	Methods: Data Pre-processing; Task Definition
	12c	D	Specify the type of model, rationale, all model-building steps, including any hyperparameter tuning, and method for internal validation	Methods: Model Selection; Knowledge Distillation for Scalable Inference
	12d	D;E	Describe if and how any heterogeneity in estimates of model parameter values and model performance was handled and quantified across clusters	Methods: Three-stage Validation (subgroup analyses by age, sex, primary pain diagnosis, clinic type; bootstrap CIs at patient level; interaction terms in pooled regression models)
	12e	D;E	Specify all measures and plots used (and their rationale) to evaluate model performance	Methods: Stage 3: Concordance Between the Scalable Lightweight Model and Patient-Reported Outcomes (AUROC, sensitivity, specificity, PPV, NPV)
	12f	E	Describe any model updating arising from the model evaluation	N/A. This is a development-phase protocol; no model updating is planned within the scope of this study.
	12g	E	For model evaluation, describe how the model predictions were calculated	N/A. This is a development study; no external validation of an existing model.
Class imbalance	13	D;E	If class imbalance methods were used, state why and how this was done, and any subsequent methods to recalibrate the model or the model predictions	Not applicable at protocol stage; class imbalance handling to be assessed during model implementation
Fairness	14	D;E	Describe any approaches that were used to address model fairness and their rationale	Methods: Three-stage Validation (pre-specified subgroup analyses including race/ethnicity, rurality; flagging of subgroups where lower CI bound for AUROC falls below cohort estimate); Discussion: Limitations
Model output	15	D	Specify the output of the prediction model and provide details and rationale for any classification and how the thresholds were identified	Methods: Primary Pain Outcomes (binary indicator, PEG domain score ≥ 7); Task Definition
Development versus evaluation	16	D;E	Identify any differences between the development and evaluation data in healthcare setting, eligibility criteria, outcome, and predictors	Methods: Training, model selection, and held-out evaluation (development and held-out test partitions drawn from the same cohort and same survey-anchored windows;

Section/Topic	Item	Dev/Eval	Checklist item	Reported on sections
				stratified by baseline pain status)
Ethical approval	17	D;E	Name the institutional research board or ethics committee that approved the study and describe the participant-informed consent or the ethics committee waiver of informed consent	Methods: Ethics Approval; Data Governance and Privacy Safeguards
**OPEN SCIENCE**				
Funding	18a	D;E	Give the source of funding and the role of the funders for the present study	Funding section
Conflicts of interest	18b	D;E	Declare any conflicts of interest and financial disclosures for all authors	Conflicts of Interest section
Protocol	18c	D;E	Indicate where the study protocol can be accessed or state that a protocol was not prepared	The present manuscript is the study protocol.
Registration	18d	D;E	Provide registration information for the study, including register name and registration number, or state that the study was not registered	This study was not registered, as it constitutes a secondary analysis of a previously conducted quality improvement evaluation (see Methods: Ethics Approval).
Data sharing	18e	D;E	Provide details of the availability of the study data	Data Availability section (data are derived from the VA CDW and CIH Therapy Patient Experience Survey; access is governed by the MOU with OPCC&CT and VA data-sharing policies; not publicly available)
Code sharing	18f	D;E	Provide details of the availability of the analytical code	Analytical code will be made available upon study completion in a public repository, subject to VA data governance constraints. To be added in the Data Availability section.
**PATIENT & PUBLIC INVOLVEMENT**				
Patient & Public Involvement	19	D;E	Provide details of any patient and public involvement during the design, conduct, reporting, interpretation, or dissemination of the study or state no involvement	Discussion: Limitations (patient representatives were not directly consulted in this secondary analysis; patient perspectives are embedded indirectly via the patient-validated psychometric instruments: PROMIS-PI, WHYMPI, Oswestry: used to construct the seed lexicon)
**RESULTS**				
Participants	20a	D;E	Describe the flow of participants through the study, including the number of participants with and without the outcome and, if applicable, a summary of the follow-up time	Results (study ongoing; cohort identification described in Methods: Study Setting and Results)

Section/Topic	Item	Dev/Eval	Checklist item	Reported on sections
	20b	D;E	Report the characteristics overall and, where applicable, for each data source or setting, including the key dates, key predictors, treatments received, sample size, number of outcome events, follow-up time, and amount of missing data	Results; cohort demographic and clinical characteristics previously published [54]
	20c	E	For model evaluation, show a comparison with the development data of the distribution of important predictors	N/A. No external evaluation cohort; all evaluation is internal via held-out partition described in Methods: Training, model selection, and held-out evaluation.
Model development	21	D;E	Specify the number of participants and outcome events in each analysis	To be reported in the final results manuscript; sample sizes for each analysis stage are pre-specified in Methods: Construct Operationalization; Training, model selection, and held-out evaluation.
Model specification	22	D	Provide details of the full prediction model to allow predictions in new individuals and to enable third-party evaluation and implementation	To be reported in the final results manuscript. Full model artifact and inference code will be released in a public repository, subject to VA data governance constraints.
Model performance	23a	D;E	Report model performance estimates with confidence intervals, including for any key subgroups. Consider plots to aid presentation	To be reported in the final results manuscript per pre-specified plan in Methods: Stage 3 (AUROC, sensitivity, specificity, PPV, NPV, F1, 95% bootstrap CIs at patient level; subgroup reporting)
	23b	D;E	If examined, report results of any heterogeneity in model performance across clusters	To be reported in the final results manuscript per pre-specified subgroup and interaction-testing plan in Methods: Stage 3
Model updating	24	E	Report the results from any model updating	N/A. No model updating planned within the scope of this study.
**DISCUSSION**				
Interpretation	25	D;E	Give an overall interpretation of the main results, including issues of fairness in the context of the objectives and previous studies	Discussion: Principal Implications; Methodological Significance; Clinical and Operational Utility
Limitations	26	D;E	Discuss any limitations of the study and their effects on any biases, statistical uncertainty, and generalizability	Discussion: Limitations
Usability of the model in the context of current care	27a	D	Describe how poor quality or unavailable input data should be assessed and handled when implementing the prediction model	Discussion: Limitations

Section/Topic	Item	Dev/Eval	Checklist item	Reported on sections
	27b	D	Discuss whether users will be required to interact in the handling of the input data or use of the model, and what level of expertise is required of users	Discussion: Clinical and Operational Utility (the framework is intended as a population health management tool operating on existing EHR data; no end-user data entry is required at the point of inference)
	27c	D;E	Discuss any next steps for future research, with a specific view to applicability and generalizability of the model	Discussion: Clinical and Operational Utility; Limitations; Conclusion
