# Supplementary material for: Extraction of Pain Severity and Functional Interference From Clinical Narratives Using Domain-Informed Large Language Models: Protocol for a Development and Validation Study
Source: JMIR Res Protoc. 2026 Oct 7;15:e96346. doi: 10.2196/96346 (PMC13645315; doi:10.2196/96346)
Supplement: Supplementary file 3 [file resprot-v15-e96346-s002.docx]

**Multimedia Appendix 2.** Shell Table – Encounter-level classification accuracy of candidate LLMs against SME annotation for each PEG domain and for binary severity level ≥7.

|  | | **SME annotation agreement** | | | | **Performance metrics** | | | |
| --- | --- | --- | --- | --- | --- | --- | --- | --- | --- |
|  |  | True negative | True positive | False negative | False positive | Precision | Recall | F1 domain | F1 overall |
| Gemma 4 E4B [44] | P |  |  |  |  |  |  |  |  |
|  | E |  |  |  |  |  |  |  |  |
|  | G |  |  |  |  |  |  |  |  |
|  | P≥7 |  |  |  |  |  |  |  |  |
|  | E≥7 |  |  |  |  |  |  |  |  |
|  | G≥7 |  |  |  |  |  |  |  |  |
| MedGemma 1.5 [45] | P |  |  |  |  |  |  |  |  |
|  | E |  |  |  |  |  |  |  |  |
|  | G |  |  |  |  |  |  |  |  |
|  | P≥7 |  |  |  |  |  |  |  |  |
|  | E≥7 |  |  |  |  |  |  |  |  |
|  | G≥7 |  |  |  |  |  |  |  |  |
