# Supplementary material for: Extraction of Pain Severity and Functional Interference From Clinical Narratives Using Domain-Informed Large Language Models: Protocol for a Development and Validation Study
Source: JMIR Res Protoc. 2026 Oct 7;15:e96346. doi: 10.2196/96346 (PMC13645315; doi:10.2196/96346)
Supplement: Supplementary file 4 [file resprot-v15-e96346-s003.docx]

**Multimedia Appendix 3.** Shell table (Partial) – Final validation reporting of Lightweight Model and survey responses.

| **PEG^a^ domain** | **Survey PROs^b^** | **ClinicalBERT^c^ model** | **NPV^d^/PPV^e^** | **AUROC^f^** |
| --- | --- | --- | --- | --- |
|  | <7, ≥7, Missing | <7, ≥7, Missing |  |  |
| **Participants with Encounters ±7 Days within Survey Date, n=3259** | | | | |
| P | 613, 2646, 0 |  |  |  |
| E | 1298, 1961, 0 |  |  |  |
| G | 1268, 1991, 0 |  |  |  |
| **Participants with Encounters ±30 Days within Survey Date, n=3718** | | | | |
| P | 714, 3004, 0 |  |  |  |
| E | 1517, 2201, 0 |  |  |  |
| G | 1499, 2219, 0 |  |  |  |
| **Participants with Encounters ±90 Days within Survey Date, n=3725** | | | | |
| P | 715, 3010, 0 |  |  |  |
| E | 1522, 2203, 0 |  |  |  |
| G | 1503, 2222, 0 |  |  |  |
| **Participants with Encounters ±180 Days within Survey Date, n=3725** | | | | |
| P | 715, 3010, 0 |  |  |  |
| E | 1522, 2203, 0 |  |  |  |
| G | 1503, 2222, 0 |  |  |  |

^a^PEG: pain, enjoyment, general activity.

^b^PRO: patient-reported outcome.

^c^BERT: bidirectional encoder representations from transformers.

^d^NPV: negative predictive value.

^e^PPV: positive predictive value.

^f^AUROC: area under the receiver operating characteristic curve.
